# Halotolerant PGPR *Stenotrophomonas maltophilia* BJ01 Induces Salt Tolerance by Modulating Physiology and Biochemical Activities of *Arachis hypogaea*

**DOI:** 10.3389/fmicb.2020.568289

**Published:** 2020-10-14

**Authors:** Ankita Alexander, Vijay K. Singh, Avinash Mishra

**Affiliations:** ^1^Division of Applied Phycology and Biotechnology, CSIR – Central Salt and Marine Chemicals Research Institute, Bhavnagar, India; ^2^Academy of Scientific and Innovative Research (AcSIR), CSIR, Ghaziabad, India

**Keywords:** peanut, saline agriculture, halotolerant bacteria, salt stress, *Stenotrophomonas*, PGPR - plant growth-promoting rhizobacteria, *Arachis hypogaea*, plant microbe interaction

## Abstract

*Arachis hypogaea* (Peanut) is one of the most important cash crops grown for food and oil production. Salinity is a major constraint for loss of peanut productivity, and halotolerant plant growth promoting bacteria not only enhance plant-growth but also provide tolerance against salt stress. The potential of halotolerant bacterium *Stenotrophomonas maltophilia* BJ01 isolated from saline-soil was explored to enhance the growth of peanut plants under salt stress conditions. Interaction of *S. maltophilia* BJ01 enhances the growth of the peanut plants and protects photosynthetic pigments under salt stress. Lower electrolyte leakage (about 20%), lipid peroxidation (2.1 μmol g^–1^ Fw), proline (2.9 μg mg^–1^ Fw) content and H_2_O_2_ (55 μmol g^–1^ Fw) content were observed in plants, co-cultivated with PGPR compared to untreated plants under stress condition. The growth hormone auxin (0.4 mg g^–1^ Fw) and total amino acid content (0.3 mg g^–1^ Fw) were enhanced in plants co-cultivated with PGPR under stress conditions. Overall, these results indicate the beneficial effect of *S. maltophilia* BJ01 on peanut plants under salt (100 mM NaCl) stress conditions. In conclusion, bacterium *S*. *maltophilia* BJ01 could be explored further as an efficient PGPR for growing legumes especially peanuts under salt stress conditions. However, a detailed agronomic study would be needed to ascertain its commercial role.

## Introduction

Soil salinity adversely affects the system of the plants at the physiological, biochemical, and molecular levels (Roy et al., 2014). Salinity causes osmotic stress, nutrient imbalance/unavailability, reduction of photosynthesis, ion toxicity, generation of reactive oxygen species (ROS), and ethylene (stress hormone) (Alexander et al., 2019a). Around the world, approximately 77 million hectares (Mha) of agricultural land is affected by salt (Arora, 2017). In India, a total of 9.38 Mha is affected by salinity, while specifically the Gujarat state has a significant share of the salinity-affected area of about 2.23 Mha (Srivastava et al., 2019). Arid and semiarid regions of the world are more affected by salinity due to inadequate rain and agricultural practices (Glick et al., 2007). Among crop plants, cereals and legumes are the most sensitive to salt. In legumes, salt affects the nodulation process and finally the nitrogen fixation (Ramana et al., 2012). Even 100 mM of salt is enough to inhibit nodule formation (Dardanelli et al., 2009). Salt creates a hindrance in Ca absorption, which in turn affects the growth of roots and root hair, hence providing an additional mechanism to hinder nodule formation (Bouhmouch et al., 2005). Peanut (*Arachis hypogaea* L.) is an important oil crop that is used for food, fodder, and industrial raw material. India in particular has been reported to have the second largest peanut production after China (Fabra et al., 2010).

To tackle the effects of salinity on crops and enhance productivity, several methods are employed, including but not limited to good agricultural practices, genetic manipulation of crops to make them resistant to salt, improvement of the agricultural soil, and irrigation water use. Application of halophilic/halotolerant plant growth-promoting bacteria in stressed soil is the most useful and environmentally friendly approach to increase the productivity and health of crops as well as enhance the soil system in the long term (Alexander et al., 2019a; Fazeli-Nasab and Sayyed, 2019). Plant roots secrete various nutrient substances (∼40% of photosynthetic products) known as root exudates, that play a significant role in the attachment and growth of various endophytic and free-living bacteria (Wang et al., 2016). Some of these bacteria enhance the plant growth and health even under stress conditions. These bacteria are known as plant growth-promoting rhizobacteria (PGPR) (Cook et al., 1995; Alexander et al., 2019b). PGPR enhance the plant growth and development in several ways, including via nitrogen fixation, phosphate solubilization, production of phytohormones, ACC deaminase activity, production of exopolysaccharide (EPS), priming the plant immunity (induced systemic resistance; ISR), acting as a biocontrol agent, and increasing the plant antioxidant enzymes that are produced under stress conditions, such as the ascorbate peroxidase (APX), the catalase (CAT), and the glutathione reductase (GR) (Kloepper et al., 2004; Arevalo-Ferro et al., 2005; Yang et al., 2009: Upadhyay et al., 2012; Bal et al., 2013; Bhargava et al., 2017; Sarkar et al., 2018).

*Stenotrophomonas* is a gram-negative, yellow-pigmented bacillus, which is a member of the gamma-Proteobacteria class (Moore et al., 1997). It is either free-living or endophytic and is associated with many plant species (Egamberdieva et al., 2016). Different species of *Stenotrophomonas* have previously been reported for their ability to promote plant growth (Ryan et al., 2009; Berg et al., 2010; Alavi et al., 2013; Singh and Jha, 2017). Egamberdieva et al. (2011) and Singh et al. (2013) have isolated *Stenotrophomonas* stains from high salinity soils. Members of this species can survive in high salt concentrations because of the production of compatible solutes, especially glucosyl glycerol (GG) and trehalose, which also help the plant to survive in harsh environmental conditions (Alavi et al., 2013). *Stenotrophomonas maltophilia* BJ01 was isolated from the rhizosphere of *Cyperus laevigatus* L., which was grown at the coastal region of Dwarka, India and submitted to the Indian marine microbial culture collection of CSMCRI, Bhavnagar with culture collection number IMMCC255 *S. maltophilia* BJ01 grew in an environment that contained up to 4% NaCl (unpublished data) and it was shown to possess the *nifH* gene (Singh et al., 2013). Quorum quenching (QQ) and antibiofilm activity of the strain has been reported, hence further supporting its ability to promote plant growth and biocontrol against various plant pathogenic bacteria. It can therefore be employed as part of a strategy that enhances plant survival under harsh growth conditions (Singh et al., 2013). We have also previously reported the nitrogen fixing ability of *S*. *maltophilia* BJ01 and its potential to promote plant growth and specifically support peanut plant growth under conditions where N_2_ is lacking (Alexander et al., 2019b).

The nitrogen fixing ability of legumes is adversely affected by soil salinity that hinders nodule formation, low microbial diversity around root which maintains the holobiome of plant hence halotolerant rhizobacterial species which can naturally survive in saline soils can be useful in agriculture especially in saline soils (Hamaoui et al., 2001; Dardanelli et al., 2008; Egamberdieva, 2011; Patel et al., 2012; Etesami and Beattie, 2018). Recently there are a few studies which shows the positive effect of PGPR on legumes under salt stress. The salt tolerance capacity of soybean was elevated when plants co-cultivated with halotolerant bacteria under 200 mM NaCl stress (Khan et al., 2019). The bacteria *Bacillus megaterium* NRCB001, *B. subtilis* subsp. *subtilis* NRCB002 and *B. subtilis* NRCB003 isolated from rice rhizosphere showed the plant growth promoting potential under salt stress (130 mM NaCl) when co-cultivated with *Medicago sativa* (alfalfa) (Zhu et al., 2020). *Bacillus megaterium* AL-18, *B. cereus* AL-19 (PGPR isolated from *Tamarix ramosissima*) improved the growth of *Phaseolus vulgaris* under salt stress (Abdelmoteleb and Gonzalez-Mendoza, 2020). This study aims to assess the plant growth-promoting attributes of a halophytic bacterium, namely *S. maltophilia* BJ01, and how these affect the growth of peanut plants under salt stress conditions.

## Materials and Methods

### Plant Material and Bacterial Interaction

Seeds of *Arachis hypogaea* cv. GG 20 were collected from the Gujarat Seed Corporation, Sihor, Gujarat, India. The seeds were surface sterilized according to the previously optimized protocol (Alexander et al., 2019b). In brief, the seeds were washed in 70% ethanol for 2 min and submerged in 0.1% HgCl_2_ for 10 min followed by washing with double autoclaved Milli-Q water 4-5 times to remove any traces of HgCl_2_. Sterilized seeds were placed in small tissue culture bottles (50 mL) containing sterilized cotton soaked with 1/2 MS (Murashige & Skoog) media in the bottom and kept in the dark for 2-3 days for germination. After 7 days of germination, seedlings of equal size were transferred to the hydroponics culture with the help of floating thermocol disks in 500 mL beaker containing 1/2 MS media. The plantlets were allowed to acclimatize for seven days. The bacterial inoculum was prepared according to the previously reported protocol Alexander et al. (2019b). In brief, For the bacterial inoculum preparation, the bacterial strain was streaked on DYGS agar plate (dextrose 1.0 g L^–1^; malate 1.0 g L^–1^; peptone 1.5 g L^–1^; yeast extract 2.0 g L^–1^; MgSO_4_.7H_2_O 0.5 g L^–1^; L-glutamic acid 1.5 g L^–1^; pH 6.0) from glycerol stock stored in -80°C and incubated for 16 hrs at 30°C, followed by subculture in 5 mL DYGS broth media overnight at 30°C and 180 rpm in an incubator shaker. The overnight grown culture was reinoculated in 150 mL of DYGS medium, and the culture was centrifuged at 4000 × *g* for 10 min once the bacterial growth reached the OD600 nm = 0.6. The supernatant was discarded, and the pellet was re-suspended in 1/2 MS media before co-cultivation with the plant.

Following acclimatization, plants were divided into four groups: (1) without bacterial inoculum and salt stress; (2) with bacterial inoculum and without salt stress; (3) without bacterial inoculum and with 100 mM salt stress; (4) with bacterial inoculum and 100 mM salt stress. All 4 sets were supplemented with 300 mL of 1/2 MS media and were grown in a culture room at 25 ± 2°C under a 16-h/8-h light/dark cycle (light intensity 170 ± 25 μmol m^–2^ s^–1^) for another 14 days. The media and inoculum in each plant were changed every seven days, and changes in morphology were recorded. The day that the plants were inoculated with the bacterium was considered to be day zero. After 14 days of stress, the root length, the shoot length, the fresh weight and the dry weight of the plants were recorded and samples were harvested for further analysis.

### Chlorophyll Estimation

Total chlorophyll contents of leaf tissues were estimated according to the method given by Arnon (1949) in which leaf tissue (100 mg) was crushed with the help of mortar pestle in 80% acetone and incubated for 6 h in the dark. This was subsequently centrifuged at 10000 × *g* and the supernatant was pooled out. Absorbance was recorded at 663 and 645 nm. Total chlorophyll contents were calculated using the following equations:

$$TotalChlorophyll=\frac{\left[\left(20.2\times Abs_{645}\right)+\left(8.02\times Abs_{663}\right)\right]\times volofthesampleinml}{weightoftissues}$$

$$Chlorophylla=\frac{\left[\left(12.7\times Abs_{663}\right)-\left(2.6\times Abs_{645}\right)\right]\times volofthesampleinml}{weightoftissues}$$

$$Chlorophyllb=\frac{\left[\left(22.9\times Abs_{645}\right)-\left(4.68\times Abs_{663}\right)\right]\times volofthesampleinml}{weightoftissues}$$

### Electrolyte Leakage

Leaves from the distal end of the primary branch were harvested and washed thoroughly with deionized water to remove surface adhered electrolytes. Samples were kept in 10 mL falcons (Eppendorf, United States) containing double distilled water and kept at room temperature on a rotary shaker for 24 h and electrical conductivity (EC) of this water (L1) was measured in μScm^–1^ using a conductivity meter (Seven Easy, Mettler Toledo, United States). These samples were autoclaved at 120°C for 20 min, cooled at room temperature (RT), and electrical conductivity (L2) was determined (Lutts et al., 1996). The electrolyte leakage was calculated by the following equation:

$$EL\left(\%\right)=\frac{L1}{L2}\times 100$$

### Membrane Stability Index

Leaves of the same age and size were harvested from the primary branch, they were washed adequately and they were kept in 10 mL vials that were placed on a shaker for 24 h. The EC was subsequently recorded. These samples were put in the water bath (Julabo) at 40°C for 30 min and cooled at RT, and the EC was measured (L_1_). The same samples were boiled off at 100°C for 20 min, and the EC (L_2_) of the cooled samples was recorded to calculate the MSI (Jha et al., 2013). The following equation was used for the calculation:

$$MSI\left(\%\right)=\left[1-\frac{L_{1}}{L_{2}}\right]\times 100$$

### Proline Content

The proline estimation was done as per Bates et al. (1973). 100 mg of plant leaf samples were crushed in liquid nitrogen and extracted in chilled sulphosalicylic acid (SSA). An equal volume of extract and ninhydrin reagent were mixed and incubated at 100°C for 1 h. After cooling the samples in ice bath, toluene was added in the reaction mixture, followed by vortexing and centrifugation. The upper phase was collected, and absorbance was taken at 520 nm. The proline content was calculated by using the standard curve of the known amount of proline.

### Total Amino Acid Content

Plant leaf samples (100 mg) were extracted with 80% chilled ethanol. This extract was treated with an equal volume of 0.2 M citrate buffer (pH 5) and 1% ninhydrin reagent. The tubes containing reaction mixture were heated at 95°C in a water bath for 15 min. After cooling, the samples were centrifuged, and the absorbance was read at 570 nm (Patel et al., 2016).

### Auxin Content

For the auxin estimation, the extract of leaf samples was prepared in 95% chilled ethanol, and the reaction was carried out further only in ice. The extract was mixed with a double amount of Salkowski reagent and was kept in the dark for 20 min. The absorbance was recorded at 535 nm (Andreae and Van Ysselstein, 1960). The total auxin amount was calculated by a standard curve drawn with the known concentration of indole acetic acid (IAA).

### Total H_2_O_2_ Contents

Extract of 100 mg leaf samples was prepared in 80% ice-cold acetone, and hydrogen peroxide was quantified by the modified method (Mukherjee and Choudhuri, 1983). The absorbance was measured at 415 nm. The total H_2_O_2_ content was calculated by a standard curve drawn with the known concentration of H_2_O_2_.

### *In vivo* Localization of Hydrogen Peroxide and Superoxide Radicals

The hydrogen peroxide and the superoxide radicals in stressed and unstressed plant samples were determined *in vivo* using a histochemical stain of 3,3- diaminobenzidine (DAB) and nitro-blue tetrazolium (NBT) respectively (Singh et al., 2016). Solutions of DAB and NBT were prepared in 10 mM phosphate buffer (pH 7.8). Fresh leaves were immersed in the freshly prepared DAB or NBT, they were kept in the dark for 2 h, and they were illuminated in white light for DAB (8 h) and NBT (1 h). The blue and brown spots that appeared on the leaves indicated *in vivo* localization.

### Lipid Peroxidation

Lipid peroxidation was estimated, according to Hodges et al., 1999 by quantifying the malondialdehyde (MDA) content. Leaf samples (100 mg) were homogenized in chilled 80% ethanol for extract preparation. The extract was divided into two sets; one set was mixed with an equal volume of thiobarbituric acid reagent (containing TBA; 1 mL of 0.5% w/v prepared in 20% w/v TCA); another set was mixed with an equal volume of TCA (20% w/v). Both sets were incubated at 95°C for 30 min, were cooled at RT, and were centrifuged at 10000 × *g* for 10 min. The optical density of the supernatant recorded at 440, 532, and 600 nm. MDA content was calculated according to the following equation:

$$A=\left[Abs_{532+TBA}-Abs_{600+TBA}\right]-\left[Abs_{532-TBA}-Abs_{600-TBA}\right]$$

$$B=\left[Abs_{440+TBA}-Abs_{600+TBA}\right]\times 0.0571$$

$$MDA\left(\mu molg^{-1}\right)=\frac{A-B}{15700}\times 10^{6}$$

### Statistical Analysis

Each group contained five plants, and the experiment was performed three times. Statistical analysis was performed by GraphPad Prism software. All data were subjected to one-way analysis of variance (ANOVA) followed by *post hoc* Tukey’s test. All values are expressed as the mean ± SE. ‘^∗^’ denotes *P* < 0.05; ‘^∗∗^’ denotes *P* < 0.01 and ‘^∗∗∗^’ denotes *P* < 0.001.

## Results and Discussion

### Interaction of *S. maltophilia* BJ01 Enhances the Growth of the Plant Under Salt Stress

**FIGURE 1:**
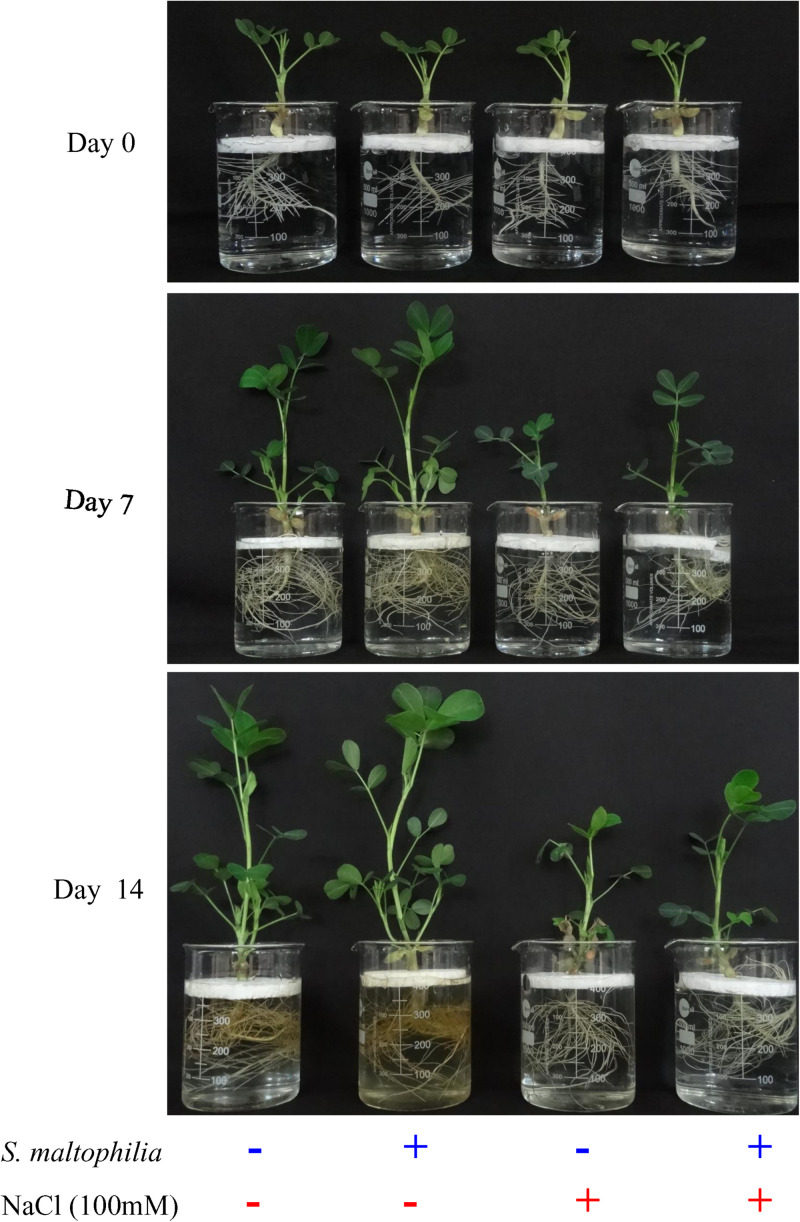
Morphological difference in inoculated and uninoculated plants. Plant grown without NaCl considered as control condition and plant under 100 mM salt considered as stressed conditions. “+” represents the presence of bacteria or NaCl, whereas “-” represents the absence of NaCl or bacteria.

**FIGURE 2:**
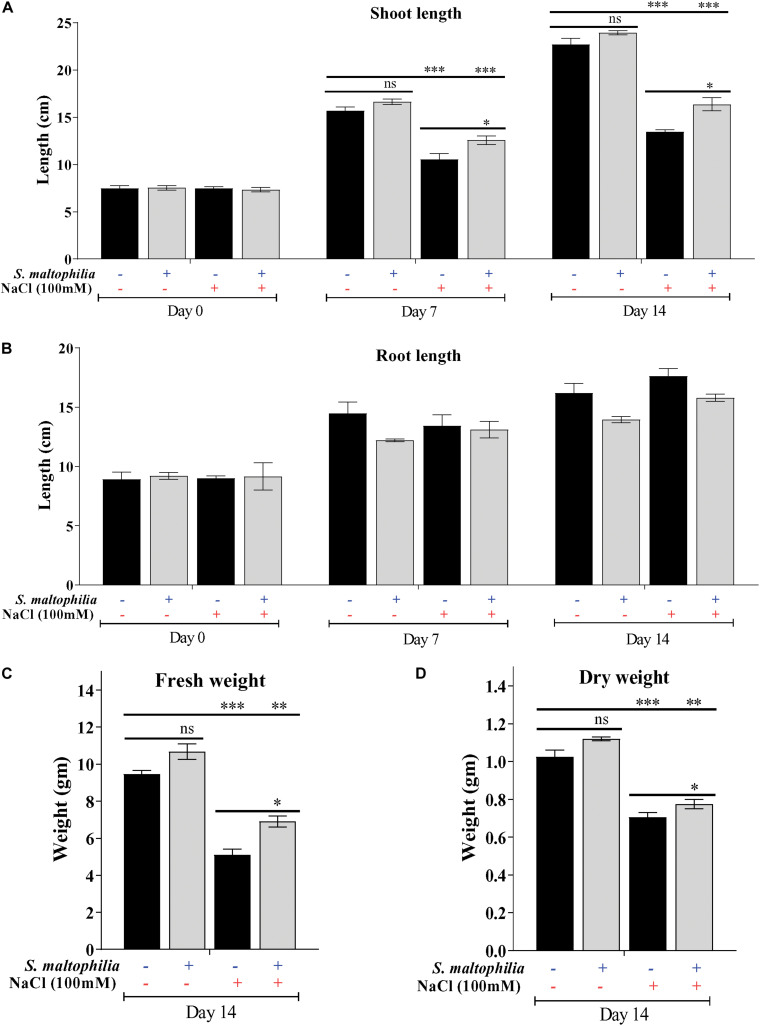
Difference in various growth parameters and comparative analysis. Shoot length **(A)**, root length **(B)** fresh weight **(C)** dry weight **(D)** of control, and stressed plants. Plant grown without NaCl considered as control condition and plant under 100 mM salt considered as stressed conditions. “+” represents the presence of bacteria or NaCl whereas “-” represents the absence of NaCl or bacteria. Bars denote means ± SE. ‘*,’ ‘**,’ ‘***’ indicate significant differences at *P* < 0.05, *P* < 0.01 and *P* < 0.001, respectively and ‘ns’ represents no significant difference.

Previously we have reported the plant growth promoting potential of *S. maltophilia* BJ01 under N_2_ starvation conditions (Alexander et al., 2019b). Here we are evaluating the potential of this bacterial strain under 100 mM salt stress condition. After the interaction of the PGPR strain, BJ01 with the plant under control condition (without salt stress) and stress condition (100 mM NaCl) plant were evaluated for their growth pattern for 14 days. Higher plant growth was observed in the plant treated with the bacteria under salt stress (Figure 1.). The shoot length of the treated plants under salt stress was significantly different from their untreated control. The shoot length of the untreated plant was about 13.4 cm whereas the shoot length of the treated plant was about 16 cm (Figure 2A). There was no significant difference in the root of the untreated and treated plants in salt stress conditions (Figure 2B). Enhanced production of auxin could be possible reason for the shoot elongation. The improved fresh weight (Fw) was observed when plants are grown with the bacteria. Under salt stress conditions, the fresh weight of the untreated plant was 5 g and the fresh weight of the treated plant was about 7 g (Figure 2C). Similarly, improved dry weight (Dw) was observed when the plant treated with bacteria. About 0.7 g and about 0.8 g of dry weight were observed in untreated and treated plants under stress conditions, respectively (Figure 2D).

For survival under abiotic stress, plants generally compromise their growth, physiology, and development because the resources like nutrients and photosynthetic byproducts are used in defense (Egamberdieva et al., 2019). In this study, we observed that the untreated plant (without bacterial interaction) under salt stress plant growth was stunned, shoot length, fresh weight, dry weight reduced drastically. On another set where plants were treated with bacterial inoculum under salt stress showed improved growth (shoot length, fresh weight and dry weight). These observations showed the role of *S. maltophilia* BJ01 in growth and development under salt stress conditions. Similar results were also reported in which PGPR showed the improved growth in crop *Solanum melongena* L, *Triticum aestivum*, and *Chenopodium quinoa* under salt stress (Fu et al., 2010; Orhan, 2016; Yang et al., 2016). Bacterial inoculation reduces the salt stress and showed the improved plant growth and phosphate uptake in *Phaseolus vulgaris* (Abdelmoteleb and Gonzalez-Mendoza, 2020). The co-cultivation of plant growth-promoting rhizobacteria also showed the improved plant growth specially the dry weight of the *Medicago sativa* (alfalfa) under salinity stress (Zhu et al., 2020).

### Photosynthetic Pigment of *Arachis hypogaea* Was Protected by *S. maltophilia* BJ01 Under Salt Stress

**FIGURE 3:**
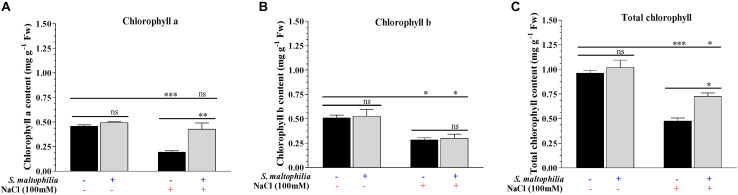
Estimation of photosynthetic pigments. Chlorophyll a contents **(A)**, chlorophyll b contents **(B)** and total chlorophyll content **(C)** of inoculated and uninoculated plants. Plant grown without NaCl considered as control condition and plant under 100 mM salt considered as stressed conditions. “+” represents the presence of bacteria or NaCl whereas “-” represents the absence of NaCl or bacteria. Bars denote means ± SE. ‘*,’ ‘**,’ ‘***’ indicate significant differences at *P* < 0.05, *P* < 0.01, and *P* < 0.001, respectively and ‘ns’ represents no significant difference.

Salt stress affects the plant cells physiologically and due to osmotic pressure, cells get dehydrated which results in stomatal closure, reduced cell growth and reduced chlorophyll content in plants (Shannon and Grieve, 1998). The peanut plants were grown under salt stress (100 mM) for 14 days. The leaves turned pale and necrosis in leaves were observed which are the sign of chlorophyll degradation and senescence. When the plants are grown with the *S. maltophilia* BJ01 under salt stress, the plant have much healthy leaves and higher chlorophyll concentration. The chlorophyll a, chlorophyll b, and total chlorophyll contents were 0.2 mg g^–1^ Fw, 0.3 mg g^–1^ Fw, and 0.5 mg g^–1^ Fw respectively in the plant without bacteria (Figures 3A–C). The chlorophyll a, chlorophyll b and total chlorophyll contents were 0.4 mg g^–1^ Fw, 0.3 mg g^–1^ Fw, and 0.7 mg g^–1^ Fw respectively in a plant grown under salt stress with bacteria (Figures 3A–C). The positive effect of rhizospheric bacteria on chlorophyll content and photosynthetic ability of host plant under saline stress condition was also reported in *Zea mays* and *Oryza sativa* (Nadeem et al., 2007; Rojas-Tapias et al., 2012; Yoolong et al., 2019). The protection of the photosynthetic pigment under salt stress was also reported in common bean (*Phaseolus vulgaris*) by rhizospheric bacteria (Abdelmoteleb and Gonzalez-Mendoza, 2020).

### *S. maltophilia* BJ01 Modulates the Plant Physiology After Interaction Under Salt Stress

**FIGURE 4:**
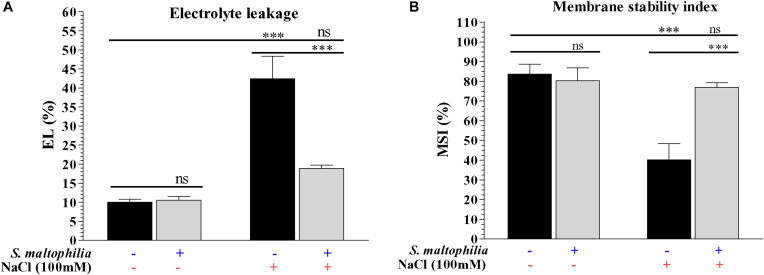
Measurement of physiological parameters. Membrane Stability Index (MSI) **(A)** and electrolyte leakage (EL) of control and stressed plants **(B)**. Plant grown without NaCl considered as control condition and plant under 100 mM salt considered as stressed conditions. “+” represents the presence of bacteria or NaCl whereas “-” represents the absence of NaCl or bacteria. Bars denote means ± SE. ‘*,’ ‘**,’ ‘***’ indicate significant differences at *P* < 0.05, *P* < 0.01, and *P* < 0.001, respectively and ‘ns’ represents no significant difference.

The plant grown under salt stress with bacteria showed reduced electrolyte leakage and high membrane integrity compared to plants grown under salt stress without bacteria. About 42% of electrolyte leakage was found in the plant grown in salt stress without bacteria and about 19% electrolyte leakage was found in the plant grown in salt stress with bacteria (Figure 4A). The membrane stability of the plant under salt stress without bacteria was about 40% whereas plants grown under salt stress with bacteria were about 77% (Figure 4B). Under salinity stress plant cells has a higher concentration of Na^+^ and Cl^–^ and low concentration of K^+^; this ionic imbalance destabilizes/damages the cell membranes (due to Ca^2+^ displacement) and causes the leakage of electrolytes from cell sap (Hussain et al., 2008). The interaction between bacteria and plants attenuate the deleterious effect on plant cells which occurs due to high salt concentration and helps cells to maintain its structure and survival. Similar results were also obtained in *Cajanus cajan* (L.), where electrolyte leakage is higher in salt condition and it reduced by the application of arbuscular mycorrhiza (Garg and Manchanda, 2009). Reduction of the electrolyte leakage in chickpea (*Cicer arietinum* L.) under salt stress by *Azospirillum lipoferum* FK1 reported by El-Esawi et al. (2019). These results are suggesting that the *S. maltophilia* BJ01 reducing the salt stress on the plant which results the improved membrane stability.

### Co-cultivation of *S. maltophilia* BJ01 Leads to the Better Biochemical Performance of *Arachis hypogaea* Under Salt Stress

**FIGURE 5:**
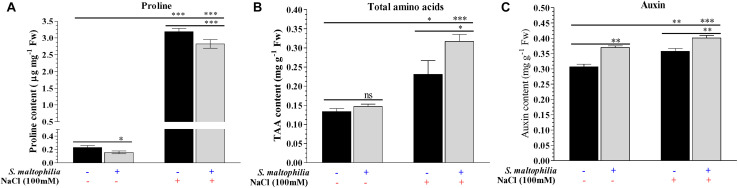
Biochemical changes in plants due to bacterial interaction. Quantification of proline **(A)**, total amino acid (TAA) **(B)** and auxin **(C)** concentration of control and treated plants. Plant grown without NaCl considered as control condition and plant under 100 mM salt considered as stressed conditions. **“+”** represents the presence of bacteria or NaCl whereas **“**-**”** represents the absence of NaCl or bacteria. Bars denote means ± SE. ‘*,’ ‘**,’ ‘***’ indicate significant differences at *P* < 0.05, *P* < 0.01, and *P* < 0.001, respectively and ‘ns’ represents no significant difference.

The plant grown with the bacteria under salt stress showed lower proline content and higher total amino acid accumulation in the plant in comparison to the control counterpart. The proline content of the plant grown under salt stress without bacteria was about 3.2 μg mg^–1^ Fw and with bacteria was about 2.9 μg mg^–1^ Fw (Figure 5A). The total amino acid concentration was quantified in plants. About 0.23 mg g^–1^ Fw was found to present in plant grown in salt stress without bacteria and about 0.31 mg g^–1^ Fw was present in the plant grown with bacteria (Figure 5B). Maintenance of turgidity and viscosity in cells is a significant challenge for plants under salt stress. To cop up with this condition plants synthesize osmolytes/osmoprotectants which help plant to survive under harsh conditions and maintain water retention inside the cells (Zulfiqar et al., 2020). Amino acids like valine, isoleucine, proline, aspartic acid, etc., act as osmoprotectants and generate in high concentration by plants under salt condition (Burg and Ferraris, 2008). Proline is one of the amino acids which acts as osmoprotectant under various abiotic stress conditions and scavenger for hydroxyl free radicles (Claussen, 2005; Peng et al., 2008). The presence of lower proline content in the plant with the bacteria reflects the role of *S. maltophilia* BJ01 to helping the plant to overcome with the salt stress. A higher amount of total amino acids (TAA) content in plants having salt stress and bacterial inoculation shows the role of bacteria further strengthen the plant system under a saline environment. Han and Lee (2005) also found that the interaction of *Glycine max* with *Bradyrhizobium japonicum* under salt stress leads to lower production of the proline. To further strengthen our finding that the plant growth promoting rhizobacteria reduces the salt stress on the plants leading the lower production of proline was also reported after interaction of halotolerant rhizobacterium *Pseudomonas koreensis* MU2 with Soybean (*Glycine max* L.) under salt stress by Adhikari et al. (2020).

The auxin production in the plant grown under salt stress without bacteria was 0.35 mg g^–1^ Fw, whereas in the plant treated with bacteria was 0.40 mg g^–1^ Fw (Figure 5C). Auxins are phytohormone which play a crucial role in the growth, development under stress conditions for plants (Egamberdieva et al., 2017). Indole-3-acetic acid (IAA) is the most common version of auxin found in plants and its concentration decreases in salt stress results in decreased growth of plants (Albacete et al., 2008). In our study, we found that the concentration of auxin decreases in salt stress without bacteria however, in plants with bacterial inoculation concentration of auxin increases. Increment in auxin concentration revealed that the bacterial interaction enhances the auxin synthesis in plants which helps plant for survival and growth under salt stress. This result is in accordance with Tiwari et al. (2011) and Noori et al. (2018).

### Reactive Oxygen Species (ROS) Buildup in *Arachis hypogea* Protected by *S. maltophilia* BJ01 Interaction Under Salt Stress

**FIGURE 6:**
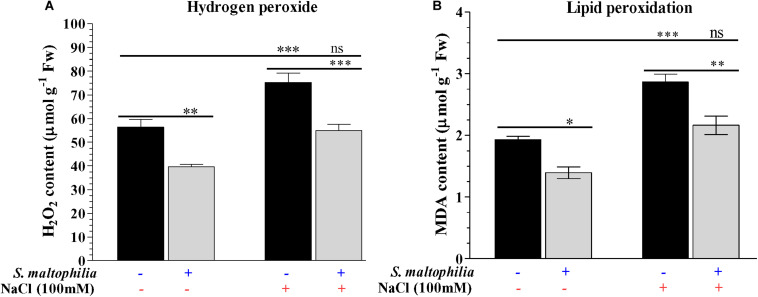
Estimation of reactive oxygen species and lipid peroxidation of the plant. Quantification of hydrogen peroxide (H_2_O_2_) **(A)** and MDA contents **(B)**. Plant grown without NaCl considered as control condition and plant under 100 mM salt considered as stressed conditions. **“+”** represents the presence of bacteria or NaCl, whereas **“**-**”** represents the absence of NaCl or bacteria. Bars denote means ± SE. ‘*,’ ‘**,’ ‘***’ indicate significant differences at *P* < 0.05, *P* < 0.01, and *P* < 0.001, respectively and ‘ns’ represents no significant difference.

**FIGURE 7:**
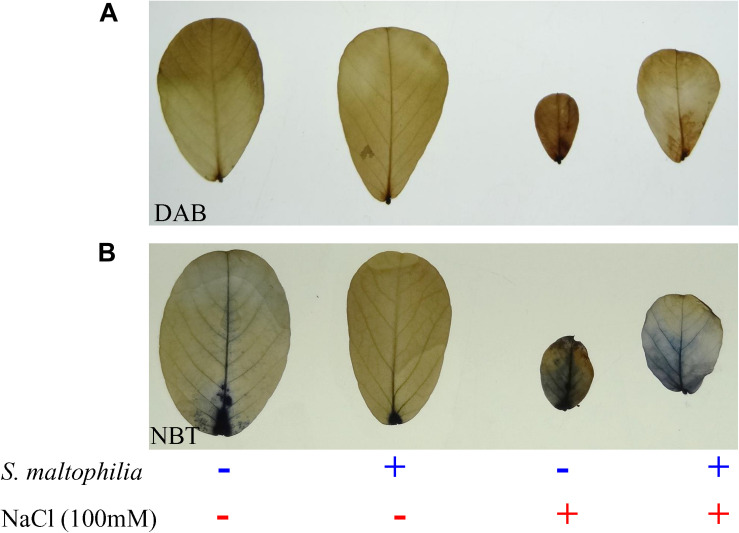
*In vivo* localization of reactive oxygen species in plant leaves. Staining of peroxide and superoxide free radicals via DAB **(A)** and NBT **(B)**. Plant grown without NaCl considered as control condition and plant under 100 mM salt considered as stressed conditions. “+” represents the presence of bacteria or NaCl, whereas “-” represents the absence of NaCl or bacteria. Bars denote means ± SE. ‘*,’ ‘**,’ ‘***’ indicate significant differences at *P* < 0.05, *P* < 0.01, and *P* < 0.001, respectively and ‘ns’ represents no significant difference.

Reduced production of hydrogen peroxide was observed in the plant grown with the bacterial in comparison to the plant grown without bacteria in the salt stress condition. About 75 μmol g^–1^ Fw H_2_O_2_ was found in the plant without bacteria under salt stress whereas 55 μmol g^–1^ Fw was measured in the plant with bacteria under stress condition (Figure 6A). These results were also supported by the *in vivo* localization of these ROS (superoxide and H_2_O_2_) in plant leaves (Figures 7A,B). In stress condition plants overproduce reactive oxygen species (ROS) which act as a signaling molecule for downstream regulation of defense mechanism; this situation called oxidative stress (Demidchik, 2015). Among the ROS, hydrogen peroxide (H_2_O_2_) is considered as the most stable molecule (Foyer and Noctor, 2009) and generate high concentration under salt stress (Singh et al., 2016). Lower H_2_O_2_ concentration in plants with bacterial inoculation in salt stress shows the beneficial effect of *S. maltophilia* BJ01 on peanut under stress conditions which reduces the oxidative stress on the plant system. Similar results were also obtained in strawberry plants by rhizobacterial treatment (Arıkan et al., 2020).

The lower production of malondialdehyde (MDA) in the plant grown with the bacteria was observed in the comparison of the plant without bacteria under salt stress. The MDA content 2.8 μmol g^–1^ Fw was measured in a plant grown without bacteria, whereas 2.1 μmol g^–1^ Fw was measured in a plant grown with the bacteria under salt stress (Figure 6B). Membrane lipids are highly reactive toward the ROS which results in lipid peroxidation and generates MDA, which is an indicator of membrane disintegration (Hodges et al., 1999; Catalá, 2006; Farmer and Mueller, 2013). More membrane damage causes more production of MDA molecules which in our case reduced by bacterial treatment in a plant under stress condition. Singh and Jha (2016) obtain similar results in wheat inoculated with halotolerant bacteria under stress conditions.

### Morpho-Physio-Biochemical Response of Plant Grown With or Without Bacteria Under Control and Stress Conditions

**FIGURE 8:**
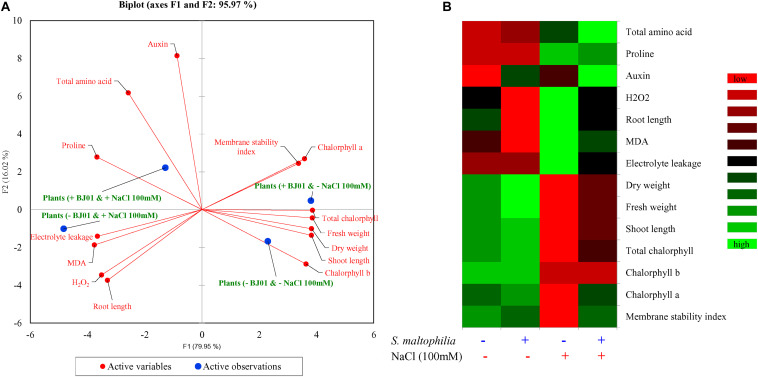
Multivariate data analyses of plant grown with or without bacteria under control and stress conditions. Principal component analysis **(A)** and integrated heat map **(B)**. Plant grown without NaCl considered as control condition and plant under 100 mM salt considered as stressed conditions. “+” represents the presence of bacteria or NaCl, whereas “-” represents the absence of NaCl or bacteria.

Principal component analysis (PCA) was carried out to extricate the response of the peanut plants under different growth conditions. The bi-plot analysis reveals the differential response of the plant under control and stress conditions when co-cultured with bacteria and without bacteria (Figure 8A). Differential responses to the variables was also observed in the integrated heat-map in different conditions of the plant growth (Figure 8B). The multivariance analysis strongly suggests that the bacterial interaction highly influence the morphology, physiology and biochemistry of the plant.

## Conclusion

In this study, the beneficial effects of *Stenotrophomonas maltophilia* BJ01 on *Arachis hypogaea* GG20 plants under 100 mM salt concentration were evaluated. Here we found that the plant growth promoting rhizobacteria isolated from halotolerant grass species can help the peanut plant to withstand the deleterious effect of salinity by supporting the plant at the morphological, physiological and biochemical level. Inhabitance in harsh conditions and nitrogen fixing ability of this bacterial strain help plants under direct salt stress. To meet up the demand for food for the growing population of the world under various abiotic stress, we need a more sustainable and environmentally friendly method. Thus, this study opens the door for the agricultural application of PGPR to overcome biotic and abiotic stress instead of chemical application. Further studies of genomic, proteomic, and metabolomics of holobiome (plant and associated microbiome) can be a beneficial intervention in this field to understand plant microbe interaction and uncover the mysteries of plant immunity and its survival.

## Data Availability Statement

The raw data supporting the conclusions of this article will be made available by the authors, without undue reservation.

## Author Contributions

AM conceived and designed the experiments. AA performed the experiments. AA, VS, and AM analyzed the data and wrote the manuscript.

## Conflict of Interest

The authors declare that the research was conducted in the absence of any commercial or financial relationships that could be construed as a potential conflict of interest.
